# Supplementary material for: Magnetogenetic cell activation using endogenous ferritin
Source: bioRxiv. 2024 Apr 25:2023.06.20.545120. Preprint. [Version 3] doi: 10.1101/2023.06.20.545120 (PMC10541561; doi:10.1101/2023.06.20.545120)
Supplement: Supplement 1 [file media-1.pdf]

Figure S1

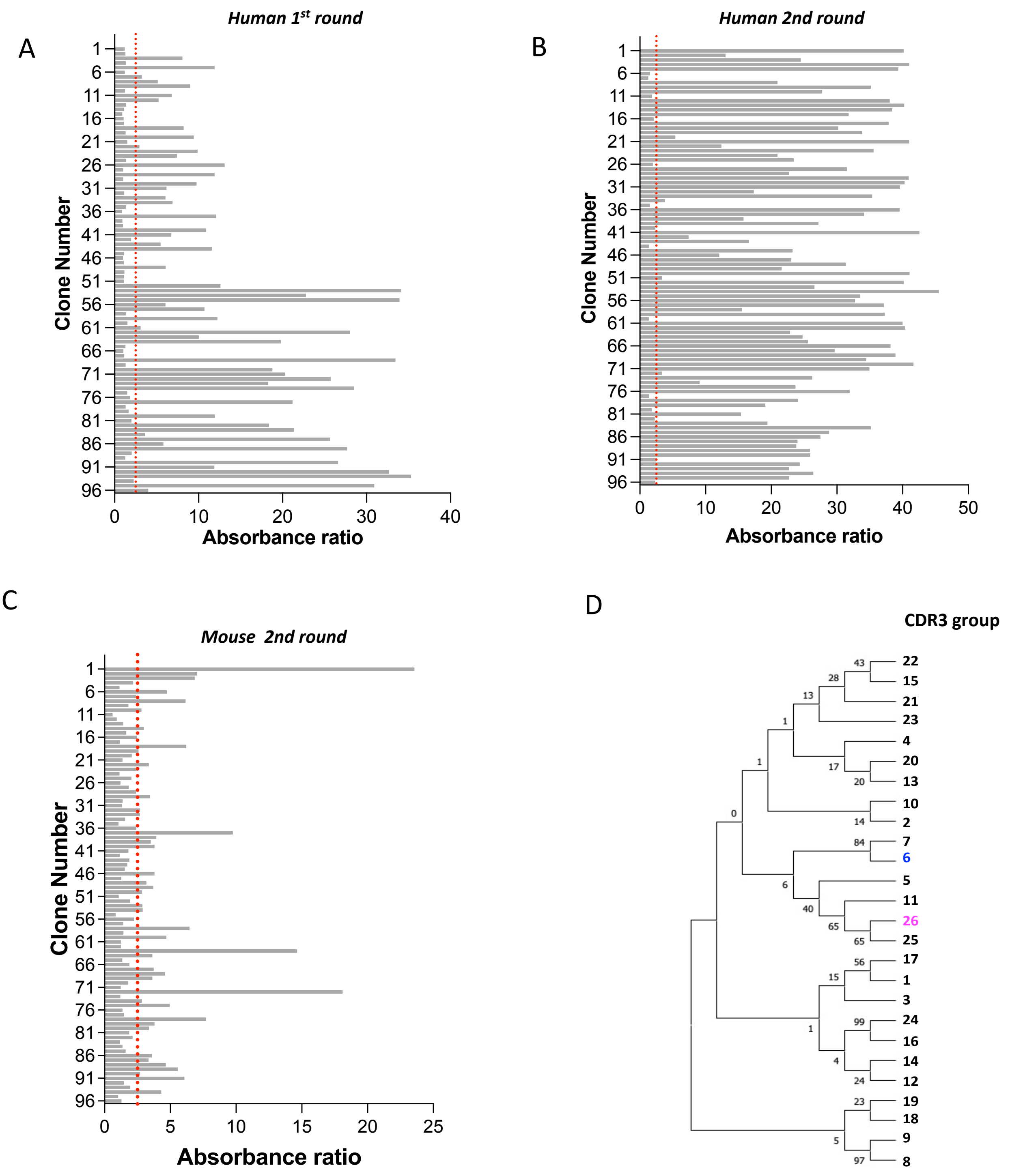
A
Human 1st round
Clone Number
Absorbance ratio
B
Human 2nd round
Clone Number
Absorbance ratio
C
Mouse 2nd round
Clone Number
Absorbance ratio
D
CDR3 group
22
15
21
23
4
20
13
10
2
7
6
5
11
26
25
17
1
3
24
16
14
12
19
18
9
8

Figure S2

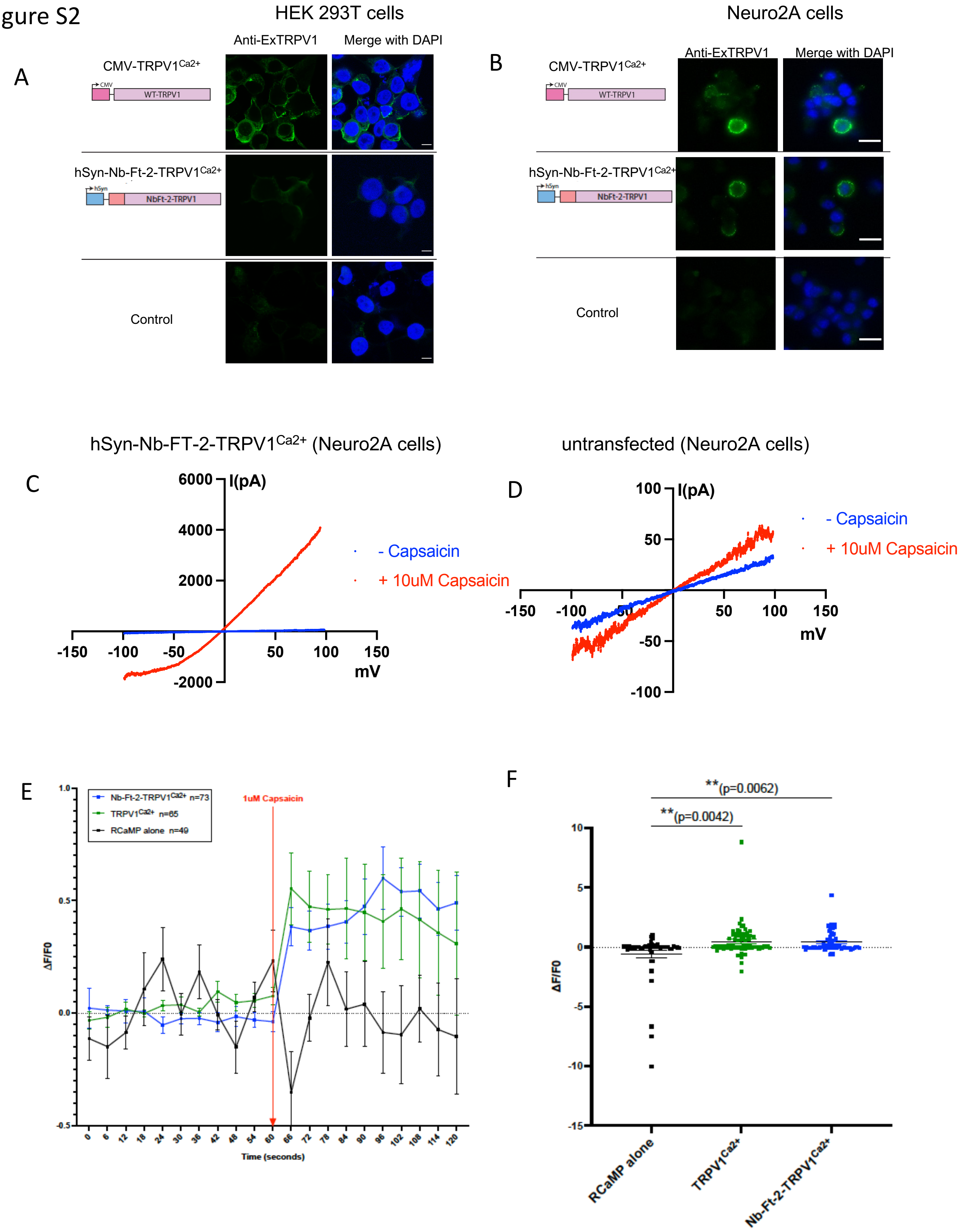
HEK 293T cells
Neuro2A cells
A
B
Anti-ExTRPV1
Merge with DAPI
CMV-TRPV1Ca2+
CMV
WT-TRPV1
hSyn-Nb-Ft-2-TRPV1Ca2+
hSyn
NbFt-2-TRPV1
Control
hSyn-Nb-FT-2-TRPV1Ca2+ (Neuro2A cells)
untransfected (Neuro2A cells)
C
D
I(pA)
mV
- Capsaicin
+ 10uM Capsaicin
6000
4000
2000
-2000
100
50
-50
-100
-150
150
E
Nb-Ft-2-TRPV1Ca2+ n=73
TRPV1Ca2+ n=65
RCaMP alone n=49
1uM Capsaicin
ΔF/F0
Time (seconds)
F
**(p=0.0062)
**(p=0.0042)
RCaMP alone
TRPV1Ca2+
Nb-Ft-2-TRPV1Ca2+

Figure S3

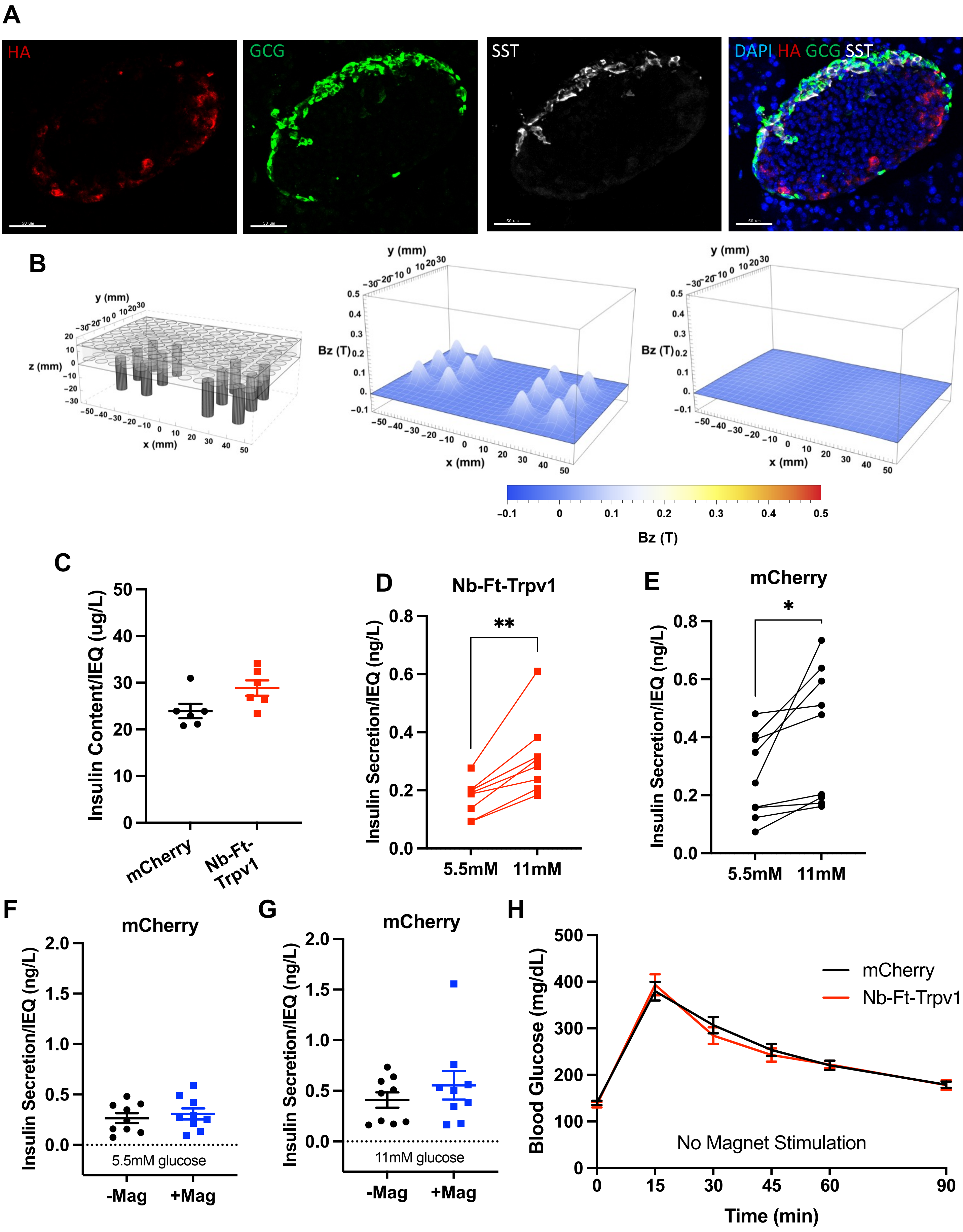

A
HA
GCG
SST
DAPI HA GCG SST
B
y (mm)
z (mm)
x (mm)
Bz (T)
C
Insulin Content/IEQ (ug/L)
mCherry
Nb-Ft-Trpv1
D
Nb-Ft-Trpv1
Insulin Secretion/IEQ (ng/L)
**
5.5mM
11mM
E
mCherry
*
F
mCherry
5.5mM glucose
-Mag
+Mag
G
mCherry
11mM glucose
H
Blood Glucose (mg/dL)
mCherry
Nb-Ft-Trpv1
No Magnet Stimulation
Time (min)
